# Pain is Associated with E-Cigarette Use and Dependence in Young Adults: Findings from Wave 7 (2022–2023) of the Population Assessment of Tobacco and Health (PATH) Study

**DOI:** 10.1007/s12529-025-10396-5

**Published:** 2025-10-03

**Authors:** Victoria E. Carlin, Joon Kyung Nam, Grant H. Ripley, Jessica M. Powers, Alexa G. Deyo, Joseph W. Ditre

**Affiliations:** 1 Center for Health Behavior Research & Innovation, College of Arts & Sciences, Syracuse University, Syracuse, NY USA; 2 Department of Psychology, College of Arts & Sciences, Syracuse University, Syracuse, NY USA; 3 Department of Psychology, University of Kansas, Lawrence, KS USA; 4 Cofrin Logan Center for Addiction Research and Treatment, University of Kansas, Lawrence, KS USA

**Keywords:** Young adults, E-cigarettes, Pain, Nicotine, Tobacco, Dependence, Co-use

## Abstract

**Background:**

Use of electronic cigarettes (e-cigarettes) among young adults (i.e., individuals aged 18 – 24) has increased substantially in recent years. Consistent with an established reciprocal model of pain and substance use, evidence demonstrates that pain increases risk for initiation and continued use of e-cigarettes, as well as dual use of e-cigarettes and other tobacco products. Meta-analytic findings also indicate that many young adults experience chronic pain (~ 12%); however, we are not aware of any prior research examining associations between pain, e-cigarette use, and dependence among young adults.

**Method:**

Data was drawn from 10,267 young adults (68% White; 50% Female) aged 18 – 24 who participated in Wave 7 (2022 – 2023) of the Population Assessment of Tobacco and Health (PATH) Study.

**Results:**

Logistic regression models indicated that moderate/severe pain (vs. no/low pain) was associated with greater odds of lifetime, current, and daily e-cigarette use. Moderate/severe pain was associated with greater odds of exclusive e-cigarette use, exclusive use of other tobacco products, and dual use of e-cigarettes and other tobacco products. Linear regression analyses further revealed that moderate/severe pain was positively associated with tobacco dependence.

**Conclusion:**

These findings suggest that moderate/severe pain may serve as a risk factor for e-cigarette use, dependence, and dual e-cigarette and other tobacco product use among young adults. Future prospective research is needed to clarify temporal precedence in the onset of moderate/severe pain and e-cigarette use among young adults. Clinical interventions focused on e-cigarette prevention and cessation among young adults with chronic pain may also be warranted.

## Introduction

The use of electronic nicotine devices (hereinafter referred to as e-cigarettes, which are the most commonly used electronic nicotine device; [1]) has increased substantially in recent years [2], particularly among young adults. Americans aged 18 to 25 are more likely to use e-cigarettes compared to other age groups, with 2023 estimates indicating that nearly a quarter (24%) used e-cigarettes in the past month [3], and more than 1 in 10 (13%) reported current, established e-cigarette use (i.e., regular or habitual usage patterns; [4]). E-cigarettes contain toxicants and irritants [5], and their use has been linked to adverse effects on respiratory and cardiovascular health [6, 7]. Dependence symptoms (i.e., cravings, tolerance, and negative reinforcement; [8, 9]) are common among people who use e-cigarettes, and more than 80% of young adults who use e-cigarettes report at least one symptom of nicotine dependence [8]. This is notable, as individuals with greater nicotine dependence engage in heavier usage patterns and are less likely to quit e-cigarette use [10, 11]. Furthermore, while some may use e-cigarettes as a healthier smoking alternative [6], the majority of e-cigarette using young adults, including 72% aged 18–20 and 53% aged 21–24, have never smoked cigarettes [12], and the use of e-cigarettes has been linked with subsequent initiation of cigarette smoking among non-smoking young adults [13, 14]. Approximately 68% of individuals who use e-cigarettes also report past 30-day use of other tobacco products [15], and individuals who engage in dual tobacco product use tend to report greater nicotine dependence [16] and are more likely to transition to exclusive use of combustible tobacco cigarettes [17], compared to those who use a single tobacco product. Thus, identifying factors associated with e-cigarette use, dependence and dual product use among young adults is essential for guiding effective prevention and cessation strategies.

The presence of pain, including chronic pain (i.e., pain lasting 3 or more months, or beyond the expected healing time; [18]), has gained increased clinical and empirical attention as an important correlate of nicotine or tobacco product use [19, 20]. A reciprocal model of pain and substance use proposes bidirectional relationships between pain and the use of nicotine and tobacco products, resulting in escalations in both pain and nicotine use [20–22]. Consistent with this perspective, meta-analytic evidence indicates that nicotine produces acute analgesic effects [23], which may amplify positive reinforcement of nicotine. Conversely, cross-sectional and prospective findings demonstrate that pain increases risk for both the initiation and maintenance of e-cigarette use among the general adult population [24, 25], and greater pain severity has been associated with greater e-cigarette dependence, perceived difficulty quitting, and negative abstinence expectancies [26, 27]. Notably, pooled random-effect prevalence data indicates that 12% of individuals aged 15–34 experience chronic pain [28], and young adults with pain experience unique challenges (i.e., the transition from pediatric to adult healthcare services, difficulty with key developmental milestones; [28, 29]) which may increase their vulnerability to engage in maladaptive coping strategies, including tobacco use [30]. Indeed, pain has been linked to both cigarette smoking [31] and the use of smokeless tobacco products [32] among young adult populations. However, there is a paucity of research examining associations between pain and e-cigarette use and dependence among young adults.

Using data from Wave 7 (2022–2023) of the Population Assessment of Tobacco and Health (PATH) Study, the present analysis sought to test the following hypotheses: among young adults, the presence of moderate to severe pain will be associated with greater odds of (a) lifetime e-cigarette use; (b) current, established e-cigarette use; (c) current, established daily e-cigarette use. Further, we hypothesized that moderate to severe pain would be associated with greater odds of exclusive e-cigarette use and dual use of e-cigarettes and other tobacco products. Among individuals who engage in current, established e-cigarette use, we hypothesized that moderate to severe pain would be associated with higher scores on a measure of e-cigarette dependence.

## Method

### Participants and Data Source

The Population Assessment of Tobacco and Health (PATH) study is an ongoing longitudinal, nationally representative cohort study of the civilian, non-institutionalized population of U.S. adults and youth, which is administered by the National Institute on Drug Abuse, the National Institutes of Health, the United States Food and Drug Administration’s Center for Tobacco Products, and Westat. The PATH study employed an address-based, stratified area-probability sample design. Data were drawn from Wave 7 (2022–2023) of the PATH study, which were collected using a mix of telephone, Web based, and in-person interviews, conducted in English or Spanish. Full details about the PATH study design and methodology can be found elsewhere [33], and data underlying the current analyses can be accessed at the National Addiction and HIV Data Archive Program: https://www.icpsr.umich.edu/web/NAHDAP/studies/36498. All respondents ages 18 and older provided informed consent. Participants aged 18–24 were classified as young adults, which is consistent with an established definition of young adulthood [34]. Further, the age variable provided in PATH public use files was categorical and included the following groupings: 18–24, 25–34, 35–44, 45–54, 55–64, and 65 +. Thus, individuals who were categorized as aged 18–24 at their Wave 7 interview were included in the current analyses.

### Measures

#### Pain Severity

Pain severity was assessed using a single item: “In the past 7 days, how would you rate your pain on average on a scale from 0 to 10, where 0 is no pain and 10 is the worst pain imaginable.” Prior research has shown associations with both past-week pain and more persistent measures of pain severity [35, 36]. Scores were dichotomized as moderate to severe pain (scores > 4/10) and no to low pain (scores ≤ 4/10) consistent with previous research using the PATH study [25, 37–40].

#### E-Cigarette Use

Lifetime e-cigarette use; current, established e-cigarette use; and daily e-cigarette use were assessed using PATH-derived variables. Lifetime e-cigarette use (0 = non-use, 1 = lifetime use) was indicated by a response of “yes” to the following question: “Have you ever used electronic nicotine products, even one or two times?” Participants met the criteria for current, established e-cigarette use (0 = non-use, 1 = current, established use) if they endorsed the following: (1) lifetime use of electronic nicotine products (responded “yes” to “Have you ever used electronic nicotine products, even one or two times?”), (2) using e-cigarettes fairly regularly in the past (responded “yes” to “Have you ever used electronic nicotine products fairly regularly?”), and (3) current use e-cigarettes on some days or every day (responded “every day” or “some days” to “Do you now use electronic nicotine products every day, some days or not at all”). Participants were categorized as engaging in daily e-cigarette use (0 = non-use, 1 = daily use) if they met the criteria for current, established e-cigarette use, and endorsed using e-cigarettes every day.

#### E-Cigarette Dependence

Dependence was measured using a tobacco dependence (TD) scale created for the PATH study [41], which assesses dependence across different nicotine and tobacco product types. The TD scale includes 16 items, drawn from the Wisconsin Inventory of Smoking Dependence Motives [9, 42] Tobacco Dependence Symptom Scale [43], and the Diagnostic and Statistical Manual, fifth edition [44], and assesses constructs such as automaticity, loss of control, negative reinforcement, craving, tolerance and withdrawal. The TD scale includes 15 items scored on a scale ranging from 1 (not true of me at all) to 5 (extremely true of me), and 1 binary item, with 0 = no, 1 = yes. Items are then summed to generate a composite TD score ranging from 15–76, with higher scores reflecting greater dependence symptoms. The TD scale has scale has been psychometrically validated as a measure of dependence across numerous tobacco products, including e-cigarettes [41], and higher TD scores have been shown to predict greater tobacco use frequency and lower odds of cessation [45]. In this sample, the internal consistency of the TD scale was excellent (*α* = 0.96).

#### Other Tobacco Product Use

Other tobacco product use was measured according to the criteria for current, established use of the non-e-cigarette tobacco products assessed by the PATH study (i.e., cigarettes, traditional cigars, cigarillos, filtered cigars, pipe, hookah, snus, smokeless tobacco, or IQOS). For tobacco cigarettes, the criteria for current, established use were (1) ever cigarette use, (2) having smoked ≥ 100 cigarettes across lifetime, and (3) current use of cigarettes every day or on some days. The criteria for current, established use of other tobacco products were (1) ever use of the tobacco product, (2) regular use of the tobacco product in the past, and (3) current some day or every day use of the tobacco product. PATH derived variables were compiled into a dichotomous other tobacco product use variable, which was coded as 0 = no other tobacco use, and 1 = current, established use of any non-e-cigarette tobacco product.

#### Dual Use of E-Cigarettes and Other Tobacco Products

Dual use of e-cigarettes and other tobacco products were compiled based on PATH derived variables. A categorical variable, indicating dual e-cigarette/other tobacco product use, was created and had following categorizations: 1) no tobacco product use, 2) exclusive e-cigarette use, 3) exclusive use of other tobacco products (i.e., cigarettes, traditional cigars, cigarillos, filtered cigars, pipe, hookah, snus, smokeless tobacco, or IQOS), and 4) dual use of e-cigarettes and other tobacco products.

#### Sociodemographic Variables

Participants reported various sociodemographic characteristics, including gender (male, female), racial identity (White, Black, other), ethnicity (Hispanic, non-Hispanic), annual household income (< $10,000, $10,000 – $24,999 $25,000 – $49,999, $50,000 – $99,999, > $100,000), and education (less than high school, GED, high school graduate, some college or associate’s degree, bachelor’s degree or beyond).

### Data Analytic Plan

All analyses were conducted using Stata Version 18.5 [46]. Missing values were present for all variables of interest, ranging from 0.02% – 8.3%. Missing data were excluded using listwise deletion, such that cases with missing values on any model variable were excluded from the relevant analysis. First, unweighted counts and weighted frequencies of sociodemographic and e-cigarette use characteristics were examined in the total sample and as a function of pain severity (i.e., no or low pain vs. moderate to severe pain). Chi-squared models were employed to test whether these proportions differed as a function of pain severity. Second, binary logistic regression models tested whether the presence of moderate to severe pain was associated with greater adjusted odds of (1) lifetime e-cigarette use; (2) current, established e-cigarette use; and (3) daily e-cigarette use. Third, a multinomial logistic regression model was used to determine whether moderate to severe pain (vs. no or low pain) was associated with a greater adjusted relative risk of exclusive e-cigarette use, exclusive use of other tobacco products, and dual use of e-cigarettes and other tobacco products, with no tobacco product use as the reference category. Fourth, the sample was limited to participants who engaged in current, established e-cigarette use, and a linear regression model tested associations between pain severity and TD scores. Given known associations with variables of interest [47–52], gender, race, ethnicity, education, and income were included as covariates in all models, and use of other tobacco products was included as a covariate in all models except for the one examining dual product use. Population and replicate weights were applied in all analyses to ensure representativeness and to account for complex sampling methods and non-response, per recommendations from the PATH study [33]. Balanced repeated replication methods were used to generate estimates, using Fay’s adjustment value of 0.3 [53].

## Results

### Sample Characteristics

The current sample included 10,267 young adults aged 18 to 24 who provided data during Wave 7 of the PATH study. Participants were predominantly White (68%), and non-Hispanic (76%), and about 50% identified as female. Approximately 14% of young adults reported moderate/severe pain. The prevalence of current, established e-cigarette use was 25% among those with moderate/severe pain and 14% among those with no/low pain. Likewise, rates of daily e-cigarette use were about twice as high among individuals with moderate/severe pain, when compared to those with no/low pain (16% vs. 8%). Unweighted frequencies and weighted percentages of sociodemographic characteristics and e-cigarette use patterns, stratified by pain status, are displayed in Table 1. Across regression models, consistent patterns of associations between sociodemographic factors and primary outcome variables emerged. Specifically, racial identity of Black or Other, Hispanic ethnicity, female gender, and higher educational attainment were generally associated with lower odds of e-cigarette use, dual use, and tobacco dependence, while current use of other tobacco products and, in some cases, lower income levels were associated with greater odds of e-cigarette use.

**Table 1:** Sociodemographic characteristics as a function of pain severity among young adults (age 18–24) at wave 7 (2022–2023) of the PATH study

	Total Sample	No or Low Pain	Moderate or Severe Pain
	*n* (%)	*n* (%)	*n* (%)
Gender^*^			
Female	5,305 (49.73)	4,462 (49.22)	843 (52.78)
Male	5,221 (50.27)	4,515 (50.78)	706 (47.22)
Race^**^			
White	6,462 (68.01)	5,605 (68.81)	857 (63.20)
Black	1,780 (14.82)	1,445 (14.08)	335 (19.30)
Other	1,690 (17.17)	1,425 (17.11)	265 (17.50)
Ethnicity^*^			
Hispanic	3,244 (23.77)	2,769 (23.31)	495 (26.50)
Past-year annual income^**^			
< $10,000	1,794 (16.92)	1,430 (15.95)	364 (22.83)
$10,000—$24,999	1,746 (16.87)	1,446 (16.17)	300 (21.10)
$25,000—$49,999	2,131 (21.40)	1,822 (21.35)	309 (21.72)
$50,000—$99,999	2,119 (22.33)	1,841 (22.59)	278 (20.79)
> $100,000	1,900 (22.47)	1,737 (23.95)	163 (13.56)
Education^**^			
Less than high school	1,099 (10.69)	845 (9.61)	254 (17.14)
GED	256 (2.68)	205 (2.49)	51 (3.80)
High school graduate	3,838 (32.39)	3,210 (31.83)	628 (35.77)
Some college or associate’s	4,067 (41.12)	3,554 (42.04)	513 (42.04)
Bachelor’s degree	1,257 (13.13)	1,154 (14.03)	103 (7.71)
Lifetime e-cigarette use^**^	5,227 (51.26)	4,331 (49.80)	896 (60.01)
Current, established e-cigarette use^**^	1,574 (15.67)	1,227 (14.17)	347 (24.71)
Current, established daily e-cigarette use^**^	930 (9.47)	708 (8.40)	222 (15.88)
Exclusive e-cigarette use^**^	1,253 (12.38)	998 (11.42)	255 (18.17)
Exclusive other tobacco product use^**^	333 (3.29)	252 (2.97)	81 (5.21)
Dual e-cigarette/other tobacco use^**^	321 (3.29)	229 (2.75)	92 (6.54)

Table lists unweighted count and weighted percentage; Weighted prevalence estimates used population weights, which used balanced repeated replication with Fay’s adjustment of 0.3; No or low pain: pain severity ≤ 4/10; Moderate to severe pain: pain severity > 4/10; Other: American Indian or Alaska Native, Asian, Native Hawaiian or Other Pacific Islander, Multiracial; GED: General Educational Development test; ^*^χ^2^ significant at *p* < 0.05, ^**^χ^2^ significant at *p* < 0.001

### Associations Between Pain Severity and Odds of E-cigarette Use

Bivariate logistic regression analyses revealed that young adults with moderate to severe pain were 1.44 times as likely to report lifetime e-cigarette use, when compared to young adults with no or low pain (*aOR* = 1.44, 95% CI [1.25, 1.67], *p* < 0.001). Young adults with moderate to severe pain (vs. no or low pain) were 1.81 times as likely to engage in current, established e-cigarette use (*aOR* = 1.81, 95% CI [1.49, 2.20], *p* < 0.001), and were 1.89 times as likely to endorse current, established daily e-cigarette use (*aOR* = 1.89, 95% CI [1.53, 2.33], *p* < 0.001; see Table 2).

**Table 2:** Binary logistic regression models of associations between pain severity and odds of 1) lifetime e-cigarette use; 2) current, established e-cigarette use; and 3) current, established daily e-cigarette use

	Lifetime e-cigarette use (Model 1)			Current established e-cigarette use (Model 2)			Current established daily e-cigarette use (Model 3)		
	*aOR*	[95% CI]	*p*	*aOR*	[95% CI]	*p*	*aOR*	[95% CI]	*p*
Pain Severity ^a^									
No/low pain	[REF]								
Moderate/severe pain	1.44	[1.25–1.67]	< 0.001**	1.81	[1.49–2.20]	< 0.001**	1.89	[1.53–2.33]	< 0.001**
Gender									
Male	[REF]								
Female	1.09	[0.99–1.20]	0.068	0.99	[0.87–1.14]	0.931	1.08	[0.91–1.29]	0.352
Race									
White	[REF]								
Black	0.55	[0.47–0.64]	< 0.001**	0.33	[0.24–0.45]	< 0.001**	0.23	[0.15–0.35]	< 0.001**
Other	0.77	[0.68–0.88]	< 0.001**	0.84	[0.70–1.00]	0.045*	0.87	[0.70–1.08]	0.202
Ethnicity									
Hispanic	[REF]								
Non-Hispanic	1.17	[1.00–1.38]	0.053	1.90	[1.57–2.32]	< 0.001**	2.55	[1.96–3.31]	< 0.001**
Past-year income									
< $10,000	[REF]								
$10,000 – $24,999	1.20	[1.02–1.41]	0.028*	1.05	[0.84–1.32]	0.660	1.44	[1.08–1.94]	0.015*
$25,000 – $49,999	1.31	[1.13–1.52]	0.001*	1.17	[0.92–1.50]	0.195	1.72	[1.25–2.38]	0.001*
$50,000 – $99,999	1.14	[0.95–1.37]	0.169	1.11	[0.86–1.44]	0.403	1.45	[1.04–2.03]	0.029*
> $100,000	1.02	[0.86–1.22]	0.787	0.85	[0.67–1.08]	0.191	1.04	[0.78–1.38]	0.783
Education ^b^									
Less than high school	[REF]								
GED	1.64	[1.04–2.59]	0.033*	1.44	[0.97–2.14]	0.068	1.68	[1.10–2.57]	0.016*
High school graduate	1.15	[0.94–1.40]	0.181	1.02	[0.80–1.30]	0.863	0.86	[0.67–1.12]	0.269
Some college/associate’s	1.26	[1.04–1.52]	0.019*	1.07	[0.82–1.38]	0.627	0.85	[0.64–1.11]	0.226
Bachelor’s or above	1.27	[1.03–1.58]	0.027*	0.67	[0.47–0.94]	0.020*	0.41	[0.27–0.63]	< 0.001**
Other tobacco product use ^c^									
No	[REF]								
Yes	11.19	[8.15–15.4]	< 0.001**	5.37	[4.31–6.70]	< 0.001**	3.34	[2.63–4.23]	< 0.001**

All models employed population weights, which used balanced repeated replication with a Fay’s adjustment of 0.3; Current, established e-cigarette use = participants who endorsed regular use of e-cigarettes on some days or daily; Current, established daily e-cigarette use = participants who met criteria for current, established e-cigarette use and report using e-cigarettes on every day; ^a^ No or low pain: pain severity ≤ 4/10; Moderate to severe pain: pain severity > 4/10; ^b^ GED: General Educational Development test; ^c^ Other tobacco product use = current, established use of cigarettes, traditional cigars, cigarillos, filtered cigars, pipe, hookah, snus, smokeless tobacco, or IQOS; * p < 0.05, ** *p* < 0.001

### Associations Between Pain Severity and Dual E-cigarette and Tobacco Product Use

Multinomial logistic regression analyses revealed that young adults with moderate to severe pain (vs. no or low pain) were 1.92 times as likely to exclusively use e-cigarettes (*RR* = 1.92, 95% CI [1.56, 2.36], *p* < 0.001), 1.94 times as likely to exclusively use other tobacco products (*RR* = 1.94, 95% CI [1.43, 2.65], *p* < 0.001), and were 2.58 times as likely to engage in dual use of e-cigarettes and other tobacco products (*RR* = 2.58, 95% CI [1.92, 3.49], *p* < 0.001; See Table 3).

**Table 3:** Multinomial logistic regression model of associations between pain severity and odds of exclusive and dual use of e-cigarettes and other tobacco products

	Exclusive e-cigarette use			Exclusive other tobacco product use			Dual e-cigarette and other tobacco product use		
	*RR*	[95% CI]	*p*	*RR*	[95% CI]	*p*	*RR*	[95% CI]	*p*
Pain severity ^a^									
No/low pain	[REF]								
Moderate/severe pain	1.92	[1.56–2.36]	< 0.001**	1.94	[1.43–2.65]	< 0.001**	2.58	[1.92–3.49]	< 0.001**
Gender									
Male	[REF]								
Female	0.96	[0.84–1.11]	0.573	0.53	[0.43–0.69]	< 0.001**	0.65	[0.46–0.91]	0.012*
Race									
White	[REF]								
Black	0.33	[0.23–0.45]	< 0.001**	0.83	[0.56–1.22]	0.340	0.29	[0.17–0.50]	< 0.001**
Other	0.80	[0.66–0.97]	0.024*	0.69	[0.46–1.02]	0.059	0.73	[0.49–1.08]	0.117
Ethnicity									
Hispanic	[REF]								
Non-Hispanic	2.05	[1.66–2.52]	< 0.001**	2.33	[1.73–3.12]	< 0.001**	2.88	[1.96–4.24]	< 0.001**
Past-year income									
< $10,000	[REF]								
$10,000 – $24,999	1.02	[0.79–1.32]	0.864	0.89	[0.62–1.29]	0.541	1.05	[0.70–1.56]	0.826
$25,000 – $49,999	1.21	[0.93–1.58]	0.151	0.98	[0.67–1.44]	0.922	0.87	[0.60–1.28]	0.472
$50,000 – $99,999	1.05	[0.79–1.41]	0.714	0.51	[0.32–0.81]	0.005*	0.75	[0.49–1.17]	0.203
> $100,000	0.79	[0.61–1.02]	0.070	0.29	[0.17–0.49]	< 0.001**	0.44	[0.28–0.70]	0.001*
Education ^b^									
Less than high school	[REF]								
GED	1.46	[0.90–2.37]	0.124	1.58	[0.80–3.13]	0.183	2.35	[1.20–4.61]	0.013*
High school graduate	0.98	[0.75–1.29]	0.869	0.57	[0.37–0.90]	0.016*	0.66	[0.40–1.06]	0.085
Some college/associate’s	0.99	[0.76–1.29]	0.963	0.33	[0.21–0.52]	< 0.001**	0.49	[0.29–0.84]	0.010*
Bachelor’s or above	0.63	[0.43–0.91]	0.015*	0.26	[0.14–0.51]	< 0.001**	0.24	[0.12–0.45]	< 0.001**

The model employed population weights, which used balanced repeated replication with a Fay’s adjustment of 0.3; Exclusive e-cigarette use = current, established e-cigarette user without use of tobacco products (i.e., cigarettes, traditional cigars, cigarillos, filtered cigars, pipe, hookah, snus, smokeless tobacco, or IQOS); Exclusive other tobacco product use = current, established use of cigarettes, traditional cigars, cigarillos, filtered cigars, pipe, hookah, snus, smokeless tobacco, or IQOS., without current, established e-cigarette use; Dual e-cigarette and other tobacco product use = both current, established e-cigarette use and current, established other tobacco product use (i.e., cigarettes, traditional cigars, cigarillos, filtered cigars, pipe, hookah, snus, smokeless tobacco, or IQOS); ^a^ No or low pain: pain severity ≤ 4/10; Moderate to severe pain: pain severity > 4/10; ^b^ GED: General Educational Development test; * p < 0.05, ** *p* < 0.001

### Associations Between Pain Severity and E-cigarette Dependence

Linear regression analyses were used to examine TD scores among participants who endorsed current, established e-cigarette use. Results indicated that the presence of moderate to severe pain (vs. no or low pain) was positively associated with TD scores (*b* = 2.33, *SE* = 1.03, *p* = 0.026; see Table 4), reflecting modestly greater e-cigarette dependence symptoms among individuals with moderate to severe pain.

**Table 4:** Linear regression model of associations between pain severity and e-cigarette dependence

	Tobacco dependence Score			
	*b*	linearized SE	*t*	*p*
Pain severity				
No/low pain	[REF]			
Moderate/severe pain	2.33	1.03	2.26	0.026*
Gender				
Male	[REF]			
Female	3.06	1.19	2.56	0.012*
Race				
White	[REF]			
Black	−10.83	1.45	−7.48	< 0.001**
Other	−0.44	1.75	−0.25	0.800
Ethnicity				
Hispanic	[REF]			
Non-Hispanic	6.24	1.30	4.78	< 0.001**
Past-year income				
< $10,000	[REF]			
$10,000 – $24,999	4.20	1.77	2.37	0.020*
$25,000 – $49,999	4.71	1.80	2.62	0.010*
$50,000 – $99,999	2.57	1.82	1.42	0.160
> $100,000	1.92	1.78	1.08	0.284
Education				
Less than high school	[REF]			
GED	2.67	2.62	0.74	0.463
High school graduate	−1.80	1.72	−1.05	0.296
Some college/associate’s	0.40	1.77	0.23	0.820
Bachelor’s or above	−0.41	2.40	−0.17	0.865
Other Tobacco Product Use				
No	[REF]			
Yes	−2.58	1.47	−1.75	0.083

The model employed population weights, which used balanced repeated replication with a Fay’s adjustment of 0.3; No or low pain: pain severity ≤ 4/10; Moderate to severe pain: pain severity > 4/10; GED: General Educational Development test; Other tobacco product use = current, established use of cigarettes, traditional cigars, cigarillos, filtered cigars, pipe, hookah, snus, smokeless tobacco, or IQOS; * p < 0.05, ** *p* < 0.001

## Discussion

The present analyses are first to examine cross-sectional associations between pain severity and e-cigarette use among a nationally representative sample of young adults at Wave 7 (2022–2023) of the PATH study. Consistent with hypotheses, the presence of moderate to severe pain was associated with greater odds of lifetime e-cigarette use; current, established e-cigarette use; and current, daily e-cigarette use among young adults. The rates of current, established e-cigarette use and daily e-cigarette use among young adults with pain were about 25% and 15% respectively, or roughly double the rates observed in young adults without no or low pain. Importantly, these results were observed even after accounting for known correlates of e-cigarette use. Previous research indicates robust associations between both male gender and other tobacco product use and greater odds of e-cigarette use among young adult populations [54], which broadly aligns with the current findings.

Consistent with prior research among the general adult population [24–27, 55], the current findings suggest that moderate to severe pain may serve as a risk factor for e-cigarette use, heavier patterns of use (i.e., daily use), and greater tobacco dependence among young adults. Furthermore, results indicated that young adults with pain were more than 2.5 times as likely to engage in dual use of e-cigarettes and other tobacco products when compared to those with no or low pain. This broadly aligns with prior work demonstrating that in the general adult population, cigarette smoking adults with pain are more likely to use other nicotine and tobacco products [24, 25], perhaps as a means to supplement or increase nicotine intake, as nicotine confers acute analgesic effects [23]. Furthermore, the current findings suggest that healthcare providers may benefit from screening for e-cigarette use and dependence among young adults receiving treatment for pain.

Targeted interventions that provide personalized feedback and psychoeducation about pain-smoking interrelations have garnered success in increasing motivation to quit smoking and cessation treatment engagement among individuals with co-occurring pain and cigarette smoking [56–58]. Among the current sample, approximately 1 in 4 young adults with pain engaged in current, established e-cigarette use, indicating that investigation of the potential clinical utility of targeted e-cigarette cessation interventions among young adults with pain is warranted. Notably, young adults are more likely to initiate use of tobacco products compared to other age groups [59], and e-cigarette use has been linked with subsequent initiation of cigarette smoking among non-smoking young adults [13, 14]. Further research should examine the utility of both e-cigarette cessation and cigarette smoking prevention efforts targeting young adults who experience pain and e-cigarette use, perhaps through interventions providing psychoeducation about interrelations between pain and tobacco product use [56–58].

This study has several limitations. First, these findings are cross-sectional, precluding causal inferences or interpretation. Second, because these analyses employed a dichotomous indicator of past-week pain severity, future research would benefit from examining interplay between e-cigarette use and more granular measures of pain severity, including the presence of chronic pain [55] and functional impairment [60]. Third, the current study did not assess cognitive-affective transdiagnostic vulnerability factors (e.g., pain-related anxiety, anxiety sensitivity, distress tolerance) which have been shown to play an explanatory role in the presence of co-occurring pain and use of nicotine/tobacco products [21, 26, 61–64]. Fourth, use of flavored e-cigarettes, which are popular among young adults and have been linked to greater e-cigarette dependence [65], were not examined. Given that previous research has shown links between pain and the use of menthol cigarettes [37], future work would benefit from examining associations between pain, nicotine dependence, and use of e-cigarettes with menthol or synthetic cooling flavors [66].

In summary, the present findings indicate that among a nationally representative sample of young adults aged 18–24, pain was associated with greater odds of e-cigarette use, dual use of e-cigarettes and other tobacco products, and greater e-cigarette dependence. These findings suggest the potential clinical utility of targeted e-cigarette prevention and cessation interventions for young adults with pain. Future research should also examine longitudinal trajectories of pain and e-cigarette use among young adults.

## Data Availability

Data underlying the current analyses can be accessed at the National Addiction and HIV Data Archive Program: [*https://www.icpsr.umich.edu/web/NAHDAP/studies/36498*](https://www.icpsr.umich.edu/web/NAHDAP/studies/36498).

## References

[1] Etzel RA, Wilson KM, Balk SJ, Farber HJ, Groner JA, Moore JE. Electronic Nicotine Delivery Systems. Pediatrics. 2015;136:1018–26. 10.1542/peds.2015-3222. 26504128

[2] Bandi P, Cahn Z, Goding Sauer A, Douglas CE, Drope J, Jemal A, et al. Trends in e-cigarette use by age group and combustible cigarette smoking histories, U.S. adults, 2014–2018. Am J Prev Med. 2021;60:151–8. 10.1016/j.amepre.2020.07.026. 33032869

[3] Substance Abuse and Mental Health Services Administration. Key substance use and mental health indicators in the United States: Results from the 2023 National Survey on Drug Use and Health (HHS Publication No. PEP24-07-021, NSDUH Series H-59). Center for Behavioral Health Statistics and Quality, Substance Abuse and Mental Health Services Administration. 2024. https://www.samhsa.gov/data/report/2023-nsduh-annual-national-report

[4] National Center for Health Statistics. National Health Interview Survey, 2023. Public-use data file and documentation. 2024. Retrieved September 30, 2025, from https://www.cdc.gov/nchs/nhis/documentation/2023-nhis.html

[5] National Academies of Sciences Engineering, Medicine. Public health consequences of E-cigarettes. Washington, DC: The National Academies Press; 2018. 10.17226/24952. 29894118

[6] Toll BA, Smith TT, King BA. Nicotine e-cigarettes: considerations for healthcare providers. Nat Med. 2024;30:1513–4. 10.1038/s41591-024-02926-7. 38627561

[7] Feeney S, Rossetti V, Terrien J. E-cigarettes—a review of the evidence—harm versus harm reduction. Tob Use Insights. 2022;15: 1179173X221087524. 10.1177/1179173X221087524. PMC8968985 35370428

[8] Lin C, Gaiha SM, Halpern-Felsher B. Nicotine dependence from different e-cigarette devices and combustible cigarettes among US adolescent and young adult users. Int J Environ Res Public Health. 2022;19:5846. 10.3390/ijerph19105846. PMC9140375 35627381

[9] Piper ME, Piasecki TM, Federman EB, Bolt DM, Smith SS, Fiore MC, et al. A multiple motives approach to tobacco dependence: the Wisconsin inventory of smoking dependence motives (WISDM-68). J Consult Clin Psychol. 2004;72:139–54. 10.1037/0022-006X.72.2.139. 15065950

[10] Vogel EA, Cho J, McConnell RS, Barrington-Trimis JL, Leventhal AM. Prevalence of electronic cigarette dependence among youth and its association with future use. JAMA Netw Open. 2020;3: e1921513. 10.1001/jamanetworkopen.2019.21513. PMC7780897 32074292

[11] Sobieski E, Yingst J, Foulds J. Quitting electronic cigarettes: factors associated with quitting and quit attempts in long-term users. Addict Behav. 2022;127: 107220. 10.1016/j.addbeh.2021.107220. PMC9112866 34979427

[12] Erhabor J, Boakye E, Obisesan O, Osei AD, Tasdighi E, Mirbolouk H, et al. E-cigarette use among US adults in the 2021 behavioral risk factor surveillance system survey. JAMA Netw Open. 2023;6:e2340859–e2340859. 10.1001/jamanetworkopen.2023.40859 PMC10625038 37921768

[13] Khouja JN, Suddell SF, Peters SE, Taylor AE, Munafò MR. Is e-cigarette use in non-smoking young adults associated with later smoking? A systematic review and meta-analysis. Tob Control. 2021;30:8–15. 10.1136/tobaccocontrol-2019-055433. PMC7803902 32156694

[14] Soneji S, Barrington-Trimis J, Wills T, Leventhal A, Unger J, Gibson L, et al. Association between initial use of e-cigarettes and subsequent cigarette smoking among adolescents and young adults: a systematic review and meta-analysis. JAMA Pediatr. 2017;171(8):788–97 Am Med Assoc 2017, 788–97. 10.1001/jamapediatrics.2017.1488 PMC5656237 28654986

[15] Rubenstein D, Pacek LR, McClernon FJ. Multiple tobacco product use conceptual framework: a 2021 update on evidence. Nicotine Tob Res. 2022;24:1208–17. 10.1093/ntr/ntac032. PMC9278823 35137194

[16] Sung H-Y, Wang Y, Yao T, Lightwood J, Max W. Polytobacco use and nicotine dependence symptoms among US adults, 2012–2014. Nicotine Tob Res. 2018;20:S88-98. 10.1093/ntr/nty050. PMC6093419 30125019

[17] Niaura R, Rich I, Johnson AL, Villanti AC, Romberg AR, Hair EC, et al. Young adult tobacco and e-cigarette use transitions: examining stability using multistate modeling. Nicotine Tob Res. 2020;22:647–54. 10.1093/ntr/ntz030. 30820566

[18] Nahin RL, Feinberg T, Kapos FP, Terman GW. Estimated rates of incident and persistent chronic pain among US adults, 2019–2020. JAMA Netw Open. 2023;6: e2313563. 10.1001/jamanetworkopen.2023.13563. PMC10189566 37191961

[19] Ditre JW, LaRowe LR, Powers JM, Hooker JE. Nicotine, tobacco smoking, and E-cigarettes. Compr. Clin. Psychol., Elsevier; 2022, p. 426–42. 10.1016/B978-0-12-818697-8.00125-4.

[20] LaRowe LR, Ditre JW. Pain, nicotine, and tobacco smoking: current state of the science. Pain. 2020;161:1688–93. 10.1097/j.pain.0000000000001874. PMC8924914 32701828

[21] Ditre JW, Zale EL, LaRowe LR. A reciprocal model of pain and substance use: transdiagnostic considerations, clinical implications, and future directions. Annu Rev Clin Psychol. 2019;15:503–28. 10.1146/annurev-clinpsy-050718-095440. 30566371

[22] Ditre JW, Brandon TH, Zale EL, Meagher MM. Pain, nicotine, and smoking: research findings and mechanistic considerations. Psychol Bull. 2011;137:1065–93. 10.1037/a0025544. PMC3202023 21967450

[23] Ditre JW, Heckman BW, Zale EL, Kosiba JD, Maisto SA. Acute analgesic effects of nicotine and tobacco in humans: a meta-analysis. Pain. 2016;157:1373–81. 10.1097/j.pain.0000000000000572 PMC4912401 27023418

[24] Powers JM, Heckman BW, LaRowe LR, Ditre JW. Smokers with pain are more likely to report use of e-cigarettes and other nicotine products. Exp Clin Psychopharmacol. 2020;28:601. 10.1037/pha0000335 PMC7220837 31724418

[25] Powers JM, Maisto SA, Zvolensky MJ, Heckman BW, Ditre JW. Longitudinal associations between pain and use of cigarettes and e-cigarettes in the population assessment of tobacco and health (PATH) study. Nicotine Tob Res. 2023;25:404–11. 10.1093/ntr/ntac197 PMC9910160 35965386

[26] Powers JM, LaRowe LR, Garey L, Zvolensky MJ, Ditre JW. Pain intensity, e-cigarette dependence, and cessation-related outcomes: the moderating role of pain-related anxiety. Addict Behav. 2020;111: 106548. 10.1016/j.addbeh.2020.106548. PMC7484173 32745941

[27] Zvolensky MJ, Garey L, Mayorga NA, Rogers AH, Orr MF, Ditre JW, et al. Current pain severity and electronic cigarettes: an initial empirical investigation. J Behav Med. 2019;42:461–8. 10.1007/s10865-018-9995-7 30488135

[28] Murray CB, de la Vega R, Murphy LK, Kashikar-Zuck S, Palermo TM. The prevalence of chronic pain in young adults: a systematic review and meta-analysis. Pain. 2022;163:e972–84. 10.1097/j.pain.0000000000002541 34817439

[29] Murray CB, Groenewald CB, De La Vega R, Palermo TM. Long-term impact of adolescent chronic pain on young adult educational, vocational, and social outcomes. Pain. 2020;161:439–45. 10.1097/j.pain.0000000000001732. PMC7001863 31651579

[30] Anastas T, Colpitts K, Ziadni M, Darnall BD, Wilson AC. Characterizing chronic pain in late adolescence and early adulthood: prescription opioids, marijuana use, obesity, and predictors for greater pain interference. PAIN Rep. 2018;3:e700. 10.1097/PR9.0000000000000700. PMC6344139 30706040

[31] Holley AL, Law EF, Tham SW, Myaing M, Noonan C, Strachan E, et al. Current smoking as a predictor of chronic musculoskeletal pain in young adult twins. J Pain. 2013;14:1131–9. 10.1016/j.jpain.2013.04.012. PMC3791150 23810151

[32] Mattila VM, Sahi T, Jormanainen V, Pihlajamäki H. Low back pain and its risk indicators: a survey of 7,040 Finnish male conscripts. Eur Spine J. 2008;17:64–9. 10.1007/s00586-007-0493-9. PMC2365533 17874146

[33] Hyland A, Ambrose BK, Conway KP, Borek N, Lambert E, Carusi C, et al. Design and methods of the population assessment of tobacco and health (PATH) study. Tob Control. 2017;26:371–8. 10.1136/tobaccocontrol-2016-052934. PMC5299069 27507901

[34] The Society for Adolescent Health and Medicine. Young Adult Health and Well-Being: A Position Statement of the Society for Adolescent Health and Medicine. J Adolesc Health. 2017;60:758–9. 10.1016/j.jadohealth.2017.03.021. 28532650

[35] O’Brien T, Breivik H. The impact of chronic pain—European patients’ perspective over 12 months. Scand J Pain. 2012;3:23–9. 10.1016/j.sjpain.2011.11.004. 29913762

[36] Von Korff M, Ormel J, Keefe FJ, Dworkin SF. Grading the severity of chronic pain. Pain. 1992;50:133–49. 10.1016/0304-3959(92)90154-4. 1408309

[37] Powers JM, Zale EL, Deyo AG, Rubenstein D, Terry EL, Heckman BW, et al. Pain and menthol use are related to greater nicotine dependence among Black adults who smoke cigarettes at wave 5 (2018–2019) of the population assessment of tobacco and health (PATH) study. J Racial Ethn Health Disparities. 2023;10:2407–16. 10.1007/s40615-022-01419-y. PMC10651305 36171497

[38] Williams CM, Mastroleo NR, Lenzenweger MF, Zale EL. Pain predicts cannabis initiation among emerging adults: results from the population assessment of tobacco and health (PATH) study. Behav Med. 2025. 10.1080/08964289.2025.2465525. 40009033

[39] Rubenstein D, McClernon FJ, Powers JM, Aston ER, Keefe FJ, Sweitzer MM. Pain is associated with exclusive use and co-use of tobacco and cannabis: findings from wave 5 (2018–2019) of the population assessment of tobacco and health study. Addict Behav. 2023;146: 107814. 10.1016/j.addbeh.2023.107814. PMC10460575 37499280

[40] Rubenstein D, Green MJ, Sweitzer MM, Keefe FJ, McClernon FJ. Bidirectional relationships between pain and patterns of cannabis and tobacco use in a US nationally representative sample. Pain. 2025;166:518–26. 10.1097/j.pain.0000000000003381. PMC11810616 39172858

[41] Strong DR, Pearson J, Ehlke S, Kirchner T, Abrams D, Taylor K, et al. Indicators of dependence for different types of tobacco product users: descriptive findings from wave 1 (2013–2014) of the population assessment of tobacco and health (PATH) study. Drug Alcohol Depend. 2017;178:257–66. 10.1016/j.drugalcdep.2017.05.010. 28675817

[42] Smith SS, Piper ME, Bolt DM, Fiore MC, Wetter DW, Cinciripini PM, et al. Development of the brief Wisconsin inventory of smoking dependence motives. Nicotine Tob Res. 2010;12:489–99. 10.1093/ntr/ntq032. PMC2861888 20231242

[43] Shiffman S, Waters A, Hickcox M. The nicotine dependence syndrome scale: a multidimensional measure of nicotine dependence. Nicotine Tob Res. 2004;6:327–48. 10.1080/1462220042000202481. 15203807

[44] American Psychiatric Association, editor. Diagnostic and statistical manual of mental disorders: DSM-5-TR^TM^. Fifth edition, text revision. Washington, DC: American Psychiatric Association Publishing; 2022. 10.1176/appi.books.9780890425787.

[45] Strong DR, Leas E, Noble M, White M, Frissell KC, Glasser A, et al. Predictive validity of the adult tobacco dependence index: findings from waves 1 and 2 of the population assessment of tobacco and health (PATH) study. Drug Alcohol Depend. 2020;214:108134. 10.1016/j.drugalcdep.2020.108134. PMC7446939 32629146

[46] StataCorp. Stata Statistical Software: Release 18. College Station, TX: StataCorp LLC; 2023.

[47] Lee J, Oh M. The moderating effect of gender on the association between E-cigarette use and smoking status: a cross-sectional study. Addict Behav. 2019;93:108–14. 10.1016/j.addbeh.2019.01.038. 30703665

[48] Assari S, Mistry R, Bazargan M. Race, educational attainment, and e-cigarette use. J Med Res Innov. 2019;4: e000185. 10.32892/jmri.185. PMC7034862 32090188

[49] Harlow AF, Stokes A, Brooks DR. Socioeconomic and racial/ethnic differences in e-cigarette uptake among cigarette smokers: longitudinal analysis of the population assessment of tobacco and health (PATH) study. Nicotine Tob Res. 2019;21:1385–93. 10.1093/ntr/nty141. PMC6751515 29986109

[50] Harlow AF, Xie W, Goghari AR, Lundberg DJ, Raquib RV, Berlowitz JB, et al. Sociodemographic differences in e-cigarette uptake and perceptions of harm. Am J Prev Med. 2023;65:356–65. 10.1016/j.amepre.2023.03.009. PMC10440280 36924804

[51] Wills TA, Knight R, Williams RJ, Pagano I, Sargent JD. Risk factors for exclusive e-cigarette use and dual e-cigarette use and tobacco use in adolescents. Pediatrics. 2015;135:e43-51. 10.1542/peds.2014-0760. PMC4279062 25511118

[52] Coleman SRM, Piper ME, Byron MJ, Bold KW. Dual use of combustible cigarettes and e-cigarettes: a narrative review of current evidence. Curr Addict Rep. 2022;9:353–62. 10.1007/s40429-022-00448-1. PMC9718538 36467719

[53] Judkins DR. Fay’s method for variance estimation. J Off Stat. 1990;6:223–39.

[54] Han G, Son H. A systematic review of socio-ecological factors influencing current e-cigarette use among adolescents and young adults. Addict Behav. 2022;135: 107425. 10.1016/j.addbeh.2022.107425. 35908319

[55] Lee J, Olayinka O, Thrul J. Association between pain and e-cigarette use stratified by cigarette smoking status: results from National Health Interview Survey (NHIS) 2019–2020. Addict Behav. 2023;140: 107625. 10.1016/j.addbeh.2023.107625. 36731225

[56] Hooten WM, LaRowe LR, Zale EL, Ditre JW, Warner DO. Effects of a brief pain and smoking cessation intervention in adults with chronic pain: a randomized controlled trial. Addict Behav. 2019;92:173–9. 10.1016/j.addbeh.2018.11.040. 30641335

[57] Ditre JW, LaRowe LR, Vanable PA, De Vita MJ, Zvolensky MJ. Computer-based personalized feedback intervention for cigarette smoking and prescription analgesic misuse among persons living with HIV (PLWH). Behav Res Ther. 2019;115:83–9. 10.1016/j.brat.2018.10.013. PMC6409188 30389092

[58] Zale EL, Maisto SA, De Vita MJ, Hooten WM, Ditre JW. Increasing cessation motivation and treatment engagement among smokers in pain: a pilot randomized controlled trial. Exp Clin Psychopharmacol. 2021;29:593–604. 10.1037/pha0000424. PMC8499853 32757595

[59] Perry CL, Pérez A, Bluestein M, Garza N, Obinwa U, Jackson C, et al. Youth or young adults: which group is at highest risk for tobacco use onset? J Adolesc Health. 2018;63:413–20. 10.1016/j.jadohealth.2018.04.011. PMC6362999 30001826

[60] Garey L, Smit T, Bizier A, Redmond BY, Ditre JW, Rogers AH, et al. Pain interference among adult dual combustible and electronic tobacco users in terms of perceived barriers for quitting. Exp Clin Psychopharmacol. 2024;32:45–53. 10.1037/pha0000660. PMC10638470 37166909

[61] LaRowe LR, Zvolensky MJ, Ditre JW. The role of anxiety-relevant transdiagnostic factors in comorbid chronic pain and tobacco cigarette smoking. Cogn Ther Res. 2019;43:102–13. 10.1007/s10608-018-9957-y.

[62] LaRowe LR, Langdon KJ, Zvolensky MJ, Zale EL, Ditre JW. Pain-related anxiety as a predictor of early lapse and relapse to cigarette smoking. Exp Clin Psychopharmacol. 2017;25:255. 10.1037/pha0000127 PMC5547034 28594195

[63] Powers JM, LaRowe LR, Lape EC, Zvolensky MJ, Ditre JW. Anxiety sensitivity, pain severity and co-use of cigarettes and e-cigarettes among adults with chronic pain. J Behav Med. 2021;44:392–401. 10.1007/s10865-021-00210-4. PMC11163874 33675503

[64] Bernstein A, Trafton J, Ilgen M, Zvolensky MJ. An evaluation of the role of smoking context on a biobehavioral index of distress tolerance. Addict Behav. 2008;33:1409–15. 10.1016/j.addbeh.2008.06.003. 18657912

[65] Li W, Davis DR, Kong G, Bold KW, Morean ME, Camenga D, et al. E-cigarette dependence and cooling flavor use are linked among youth. Drug Alcohol Depend. 2024;260:111325. 10.1016/j.drugalcdep.2024.111325. PMC11612924 38744108

[66] Leventhal AM, Tackett AP, Whitted L, Jordt SE, Jabba SV. Ice flavours and non-menthol synthetic cooling agents in e-cigarette products: a review. Tob Control. 2023;32:769–77. 10.1136/tobaccocontrol-2021-057073. PMC9613790 35483721

